# Transcriptomic Profile of Blood–Brain Barrier Remodeling in Cerebral Amyloid Angiopathy

**DOI:** 10.3389/fncel.2022.931247

**Published:** 2022-06-22

**Authors:** Muyu Situ, Ali Francisco Citalan-Madrid, Svetlana M. Stamatovic, Richard F. Keep, Anuska V. Andjelkovic

**Affiliations:** ^1^Neuroscience Graduate Program, University of Michigan Medical School, Ann Arbor, MI, United States; ^2^Department of Pathology, University of Michigan Medical School, Ann Arbor, MI, United States; ^3^Department of Neurosurgery, University of Michigan Medical School, Ann Arbor, MI, United States; ^4^Department of Molecular and Integrative Physiology, University of Michigan Medical School, Ann Arbor, MI, United States

**Keywords:** blood-brain barrier, cerebral amyloid angiopathy, vasculopathy, inflammation, permeability, aging, microglia, microbleeds

## Abstract

Cerebral amyloid angiopathy (CAA) is a small vessel disease characterized by amyloid β (Aβ) peptide deposition within the walls of medium to small-caliber blood vessels, cerebral microhemorrhage, and blood–brain barrier (BBB) leakage. It is commonly associated with late-stage Alzheimer’s disease. BBB dysfunction is indicated as a pathological substrate for CAA progression with hyperpermeability, enhancing the extravasation of plasma components and inducing neuroinflammation, further worsening BBB injury and contributing to cognitive decline. Although significant effort has been made in defining the gene mutations and risk factors involved in microvascular alterations with vascular dementia and Alzheimer’s disease, the intra- and intercellular pathogenic mechanisms responsible for vascular hyperpermeability are still largely unknown. The present study aimed to elucidate the transcriptional profile of the cerebral microvessels (BBB) in a murine model with CAA vasculopathy to define potential causes and underlying mechanisms of BBB injury. A comprehensive RNA sequencing analysis was performed of CAA vasculopathy in Tg-SwDI mice at 6 and 18 months in comparison to age-matched wildtype controls to examine how age and amyloid accumulation impact the transcriptional signature of the BBB. Results indicate that Aβ has a critical role in triggering brain endothelial cell and BBB dysfunction in CAA vasculopathy, causing an intense proinflammatory response, impairing oxidative metabolism, altering the coagulation status of brain endothelial cells, and remodeling barrier properties. The proinflammatory response includes both adaptive and innate immunity, with pronounced induction of genes that regulate macrophage/microglial activation and chemokines/adhesion molecules that support T and B cell transmigration. Age has an important impact on the effects of Aβ, increasing the BBB injury in CAA vasculopathy. However, early inflammation, particularly microglia/macrophage activation and the mediators of B lymphocytes’ activities are underlying processes of BBB hyperpermeability and cerebral microbleeds in the early stage of CAA vasculopathy. These findings reveal a specific profile of the CAA-associated BBB injury that leads to a full progression of CAA.

## Introduction

Cerebral small vessel disease (CSVD) refers to a syndrome of clinical, cognitive, neuroimaging, and neuropathological findings in small caliber blood vessels (arterioles, capillaries, small veins, and venules) that preferentially occurs in certain brain regions. These include cortex, basal ganglia, thalamus, cerebellar white matter, brainstem, and also peripheral white matter and leptomeningeal arteries (Richard et al., 2010; Thal et al., 2015; Vinters, 2015). These cerebrovascular alterations are a major cause of stroke (20% worldwide) and are one of the most common causes of vascular and mixed dementia [vascular dementia (VaD) and Alzheimer’s disease (AD); Cordonnier et al., 2006; Kovari et al., 2013; Regenhardt et al., 2020]. The “epicenter” of the pathological process is the neurovascular unit (NVU), a structure composed of barrier-endowed endothelial cells and their basement membrane, as well as adjacent astrocytes, pericytes, microglial, neurons, and extracellular matrix. NVU dysfunction can lead to impaired vascular reactivity, barrier breakdown, and progressive brain injury.

Cerebral amyloid angiopathy (CAA) is the most common form of CSVD found in patients with dementia and Alzheimer’s disease (AD) (80–100% of cases), as well as in the elderly population without dementia (45% of asymptomatic adults over 80 years old; Kovari et al., 2013; Charidimou et al., 2018; Bravo et al., 2021). CAA is characterized by progressive cerebrovascular amyloid deposition mainly in the leptomeningeal and cortical vessels of the cerebral lobes and cerebellum. Two forms of CAA are recognized: hereditary (rare) due to mutations in amyloid precursor protein, such as APP [e.g., KM670/671NL (Swedish), E693Q, APP D694N (Iowa)], and sporadic (idiopathic) commonly present in AD and elderly asymptomatic adults (Haglund et al., 2006; Viswanathan and Greenberg, 2011; Kanekiyo et al., 2012; Ghiso et al., 2014; Yamada, 2015; Bravo et al., 2021). Sporadic CAA can be classified into two subtypes. Type 1 involves predominantly cortical and leptomeningeal capillaries, as well as cortical arteries and arterioles that have a fibrillar amyloid deposition accompanied by a localized robust neuroinflammatory response and perivascular phospho-tau protein accumulation. In contrast, Type 2 involves only larger caliber vessels and predominantly soluble amyloid (Bell and Zlokovic, 2009; Yamada, 2015; Hondius et al., 2018). The capillary form of CAA is predominantly associated with vasculopathies, which represent the pathological basis for developing CAA-related cerebrovascular disorders like micro- and macro-hemorrhage, ischemic stroke, and dementia (Bell and Zlokovic, 2009; Yamada, 2015; Hondius et al., 2018).

Two steps are considered critical in CAA pathogenesis: (1) cerebrovascular amyloid deposition and (2) vascular injury including oxidative stress, mitochondrial failure, and blood–brain barrier permeability changes (Xiong et al., 2009; Carrano et al., 2011; Viggars et al., 2011; Hartz et al., 2012). Although the cause and mechanism of amyloid deposition in the perivascular space and vessel wall in CAA are still not fully defined, evidence suggests that diminished clearance of Aβ by brain endothelial cells plays a role. This can be due to genetic factors [e.g., mutations in *ApoE* ε2 or ε4 allele, presenilin 1 (PS1), α1-antichymotrypsin (ACT), neprilysin, low-density lipoprotein-receptor related protein (LRP-1), angiotensin-converting enzyme (ACE)], as well as non-genetic factors (e.g., hypertension) where dysfunctional receptor system, RAGE/LPR1, causes reduced clearance of amyloid, leading to increase Aβ accumulation (Xiong et al., 2009; Carrano et al., 2011; Viggars et al., 2011; Hartz et al., 2012). Vessel wall Aβ deposition may trigger a series of events in brain endothelial cells (oxidative stress, inflammatory phenotype, formation of ion-like channels, and cell toxicity) and the perivascular space [monocyte/macrophage activation, intense inflammatory response, extracellular matrix protein degradation by metalloproteinases (MMPs), pericyte detachment, and loss of neuronal endings], which could impair autoregulation and cause microvessels disruption leading to progressive deterioration of vessel architecture and function (Bell and Zlokovic, 2009; Carrano et al., 2011; Hartz et al., 2012; Sagare et al., 2013; Sochocka et al., 2013; Weekman and Wilcock, 2016; Hondius et al., 2018).

Several lines of evidence suggest that diminished BBB integrity and persistent brain endothelial dysfunction have a central role in CAA pathogenesis, particularly in AD (Carrano et al., 2011; Hartz et al., 2012; Tanifum et al., 2014). In the capillary form of CAA, clinical studies have shown that brain endothelial cells “suffer” metabolic damage with reduced glucose transporter 1 expression, increased pinocytotic vesicles, and decreased mitochondria number (Todd et al., 2016; Abner et al., 2020; Zhou et al., 2022). At the BBB, loss of brain endothelial tight junction (TJ) proteins, namely occludin, claudin-5, and ZO-1, has been reported (Hartz et al., 2012; Magaki et al., 2018; Zhou et al., 2022). Among the mechanisms underlying increased permeability, oxidative stress and activation of MMP-2 and -9, as well as proinflammatory cytokines are indicated as having roles (Hernandez-Guillamon et al., 2012; Todd et al., 2016; Grand Moursel et al., 2018; Sakai et al., 2021). A compromised BBB may fuel the ongoing process of amyloid deposition, cause entry of blood-borne compounds, and induce inflammation that can significantly contribute to brain endothelial cell dysfunction and capillary deterioration. However, targeting any of these factors (e.g., MMP, inflammation) has produced a limited effect, pinpointing that some other mechanism and/or factors could be involved in brain endothelial injury in CAA.

The present study addresses the cellular and molecular events that alter the BBB and NVU in CAA. It specifically emphasizes the transcriptional profile of BBB injury with CAA and how it depends on age and amyloid deposition.

## Materials and Methods

### Animals

All experimental procedures were approved by The Institutional Animal Care and Use Committee, University of Michigan. Experiments were performed on male mice from the following groups: Tg-SwDI [genetic background C57BL/6, Tg(Thy1-APPSwDutIowa)BWevn, Jackson Laboratory and MMMRC, Swedish/Dutch/Iowa mutant of amyloid precursor protein (APP) under control of the mouse Thy1.2 promoter] at 6 and 18 months, and young (6 months) and aged (18 months) wildtype mice, strain C57BL/6 (Charles River and National Institute of Aging colony).

### Magnetic Resonance Imaging

Magnetic resonance Imaging (MRI) was performed using a 7.0T Agilent MR scanner (horizontal bore, Agilent, Palo Alto, CA, United States). During imaging, mice were anesthetized with 1.75% isoflurane/air mixture and a body temperature maintained at 37°C using forced heated air. Axial T2-weighted images were acquired using a fast spin-echo sequence with the following parameters: repetition time (TR)/effective echo time (TE), 4,000/60 ms; echo spacing (ESP), 15 ms; the number of echoes, 8; field of view (FOV), 20 × 20 mm; matrix, 256 × 128; slice thickness, 0.5 mm; the number of slices 25. T2*-weighted gradient-echo images were acquired using the same slice package with the following parameters: TR/TE of 319/6 ms, flip angle 20°. For BBB permeability studies, T1-weighted spin-echo images were acquired pre- and post-gadolinium contrast injection [*gadolinium* with diethylenetriaminepentaacetic acid (Gd-DTPA), 0.5 mmol, BioPAL] using the same slice package as above and with a TR/TE of 750/9 ms, ESP 9 ms, RARE factor 2, total experimental time for each T1map 192 s. For MRI analysis, all images were first evaluated for adequate signal-to-noise ratio, presence of significant motion or other artifacts, and consistency of the sequence parameters. The total number of microbleeds per mice was determined from T2* scans taking into consideration the volume of the lesion. The minimal size of the lesion was 1 voxel (10 μm^3^). MRI data were collected in saturation recovery mode for generating T1map and converted to R1 map for calculating DR1 over time. For BBB permeability, an influx rate constant for Gd-DTPA (*K*_*i*_; min^–1^) was calculated using the Patlak Model and an established published protocol (Knight et al., 2009; Nagaraja et al., 2011).

### *In vivo* Permeability Assay

*In vivo* BBB integrity was assessed by determining the influx rate constants (*K*_*i;*_ units μl/g/min) of sodium fluorescein (376 Da), Inulin (5 kDa), and Texas Red-dextran (20 kDa) entry into the brain after intravenous administration as described previously (Sladojevic et al., 2014; Stamatovic et al., 2019).

### Immunofluorescence and Ce3D Clearing Tissue

Tg-SwDI and corresponding wildtype (WT) controls were intracardially perfused with PBS to eliminate circulating blood cells and the brains were removed and fixed with BD Cytofix reagent (BD Biosciences, San Jose, CA, United States) containing paraformaldehyde for 24 h at 4°C. After fixation, brains were washed twice for 30 min in washing buffer (0.3% Triton X-100, 0.5% 1-thioglycerol), embedded in 5% agarose, and vibratome sliced (250 μm-thick slices). Aβ plaques were visualized by Amyloid-Glo RTD reagent (Biosensis) following a modified protocol from the manufacturer. Briefly, slices were dried with 70% ethanol (5 min), rinsed with water (2 min), and incubated with Amyloid-Glo RTD reagent for 2 h at room temperature. After incubation, slides were rinsed with 0.9% saline solution followed by rinsing in distilled water for 15 s. Amyloid-Glo stained-slides were incubated with Dylight-488 labeled tomato lectin (Vector Laboratories, Newark, CA, United States) diluted in freshly prepared blocking buffer containing 1X BD Perm/Wash buffer (BD Biosciences, San Jose, CA, United States) and supplemented with 0.3% Triton X-100, 1% BSA and 1% horse serum.

For immunofluorescence staining, brain slices were incubated with mouse anti-claudin 5-Alexa Fluor 488 (Invitrogen, Carlsbad, CA, United States) and mouse anti-ZO-1-Alexa Fluor 647 (Invitrogen, Carlsbad, CA, United States) antibodies to visualize the tight junction complex. Additionally, mouse anti-GFAP (Sigma-Aldrich, St. Louis, MO, United States) and rabbit anti-Iba1 (Synaptic System) antibodies were used to label astrocytes and microglia, respectively. Following incubation with primary antibodies, samples were incubated with corresponding secondary antibodies, goat anti-mouse labeled with Alexa Fluor 647, and goat anti-rabbit labeled with Alexa Fluor 488 (both from Invitrogen, Carlsbad, CA, United States). Finally, blood vessels were stained with Dylight-594 labeled tomato lectin (Vector Laboratories, Newark, CA, United States) diluted in blocking buffer. After staining, brain slices were processed for clearing using a clearing-enhanced 3D (Ce3D) protocol (Li et al., 2019). Briefly, brain slices were cleared with Ce3D clearing solution (final concentration: 20% *N*-methylacetamine, 80% Histodenz, 0.001% Triton X-100, 0.005% of 1-thioglycerol). Brain slides were incubated with gradually increasing concentrations of Ce3D clearing solution: 30, 50, 70, 80, 90, and 100%. Following clearing, brain slices were mounted onto a no. 0 glass-bottom dish. Images (optical sections, 1 μm interval) were obtained on spinning disk confocal microscopy (Nikon Instruments, Melville, NY, United States) with a 40X water immersion objective, using constant laser intensities and pinholes through the samples.

Images analysis was done using Imaris^®^ (Bitplane Inc, South Windsor, CT, United States), Image J (NIH) software, and in-house written macros. For all image analysis, the tomato lectin fluorescence intensity signal was manually adjusted to create a 3D surface mask for every z-stack image to isolate brain microvessels using Imaris. The coverage of Aβ was determined as the absolute number and volume (μm^3^) of spots per z-stacks using an in-house generated macros on Image J to detect the overlap between a 3D surface mask of Aβ spots and the delineated perivascular space of a 3D surface mask of brain microvessels. Similarly, after background subtraction and deconvolution by ImageJ, z-stacks images were used to analyze the colocalization between claudin-5 and ZO-1. For colocalization, the overlapping of the fluorescence signal of these junction proteins falling into the created 3D mask surface of the blood vessel was determined by using in-house written macros. Pearson correlation coefficient (PCC) and Mander’s overlapping coefficient (MOC) were calculated to determine the fraction of fluorescence signal of claudin-5 overlapping the fluorescence signal ZO1 (MOC1) and vice versa (MOC2). For each analyzed group (*n* = 3 per group), a total of 15 z-stack images were analyzed in both the cortex and thalamus.

### Microvessel Isolation

Brain microvessels isolation from Tg-SwDI mice and C57BL/6 mice was performed using an already established protocol (Lee et al., 2019; Sladojevic et al., 2019). Briefly, brain tissue was minced in Dulbecco’s Phosphate Buffered Saline and homogenized gently in a Dounce glass homogenizer. Myelin was removed by centrifugation in a 15% Dextran solution (Dextran MW ∼70,000, Sigma Aldrich, St. Louis, MO, United States).

### RNA Sequencing and Bioinformatics Analysis

Total RNA was isolated using the Trizol/chloroform extraction method. RNA concentration, purity, and integrity were evaluated using Agarose Gel Electrophoresis and Agilent Bioanalyzer RNA 6000 Kit. Only RNA samples with RNA integrity number > 8 were used for library preparation. Total RNA-Seq library construction used the Illumine NovaSeq 600 platform. Total RNA was rRNA depleted followed by enzymatic fragmentation, cDNA synthesis, and double-stranded cDNA purification. Each RNA-Seq group contained three biological replicates.

The raw sequencing library was further subjected to sequencing analysis. Reads were trimmed using Cutadapt v2.3, and FastQC (v0.11.8) was used to ensure data quality. Reads were mapped to the reference genome GRCm38 (ENSEMBL) using STAR v2.7.3a and genes were assigned count estimates with RSEM v1.3.2 (Li and Dewey, 2011; Dobin et al., 2013). Alignment options followed ENCODE standards for RNA-seq (Dobin et al., 2013). FastQC was used in an additional post-alignment step to ensure that only high-quality data were used for expression quantitation and differential expression. Log-fold change shrinkage was applied to RNA-seq data using apeglm method in R. For RNA-seq data, differentially expressed genes were determined by DESeq2 R package with a cutoff of *p* < 0.05 and abs (log2 fold change) > 0.58. Using ENSEMBL from the org.Mm.eg.db, R package of Bioconducter, and the cluster Profiler R package, gene ontology (GO) functional classification and enrichment analyses were done to identify terms that were significantly enriched in the list of differentially expressed genes. Heatmaps were generated in R using the heatmap package. Gene set enrichment analysis was done using cluster Profiler R package for gene ontology terms using ranked genes by log2 Fold Change values. GSEA results were imported into the Enrichment Map application on Cytoscape using the RCy3 package. The enrichment map shows GO terms with *p* < 0.05, and edges in the enrichment map represent GO terms with a similarity coefficient of 0.3 using the overlap coefficient index.

### Real-Time Polymerase Chain Reaction

Total RNA from mouse brain microvessels was prepared using TRIzol (Thermo Fisher Scientific, Carlsbad, CA, United States). Single-strand cDNA was synthesized using SuperScript II RT (Thermo Fisher Scientific, Carlsbad, CA, United States). Quantitative PCR reactions were performed with SYBR Green PCR kit Master Mix and analyzed with 7500 Real-Time PCR system (Applied Biosystem, Bedford, MA, United States). Fold-changes in expression were calculated with an adaptation of the ΔΔCt method using β-actin as the reference gene.

### Protein Array

A Mouse Inflammatory Antibody Array kit (RayBiotech Inc, Peachtree Corners, GA, United States) was used to simultaneously detect and semi-quantify 42 cytokines/chemokines in isolated microvessels from all experimental groups. Densitometric analyses were performed using ImageJ software using Protein array analyzer plugins, and the obtained data were analyzed using Excel-based software tools provided by manufacturer.

### Western Blot

Isolated brain microvessels were washed with PBS and then lysed in RIPA buffer. Western blotting was performed with anti-claudin-5, -ZO-1, -IL6, -β-actin, and -GADPH antibodies (Cell Signaling Technology, Danvers, MA, United States). Immunoblots were exposed to secondary antibodies, either anti-mouse- or anti-rabbit-HRP conjugated antibody (BioRad), and visualized with a chemiluminescent HRP substrate kit and analyzed using Image J software.

### Enzyme-Linked Immunosorbent Assay

Isolated brain microvessels were assayed for CCL9, CCL9, and CXCL5 levels by Enzyme-linked immunosorbent assay (ELISA) using the mouse CCL9, CCL11, and CXCL5 Quantikine kit (R&D System Inc., Minneapolis, MN, United States) according to the manufacturer’s protocol.

### Statistics

Analyses were performed using GraphPad Prism 9.0. Unpaired, two-tailed Student’s test and one-way analysis of variance (ANOVA), with Tukey *post hoc* tests, were used to test group-level differences. The data were presented as value ± SD, and *p* < 0.05 was regarded as statistically significant.

## Results

**FIGURE 1:**
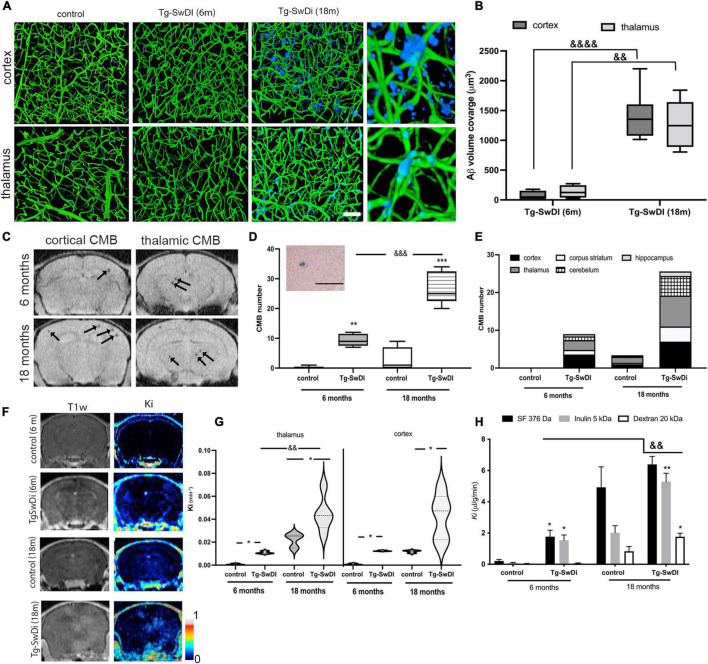
**(A)** Amyloid accumulation around the brain microvasculature in cortex and thalamus of Tg-SwDi mice (6 and 18 months old) and aged wild type mice (control; 18 months old). Blood vessels were visualized by Tomato lectin Alexa488 (green) and amyloid deposition with Amyloid Glo (blue). The images represent maximal intensity projection at 250 μm. Magnified 3-D view of the boxed region showed the localization of the Aβ deposit around blood vessels in the cortex and thalamus. Scale bar 100 μm. **(B)** Box and whisker plot represents semi-quantitation of the blood vessel Aβ coverage (μm^3^). Analysis of two regions (cortex and thalamus) showed a significant difference in Aβ deposition in Tg-SwDi mice (18 months old) in both regions compared to young Tg-SwDi mice (6 months old). No Aβ accumulation was present in any region in corresponding age-matched mouse brains. Values are from 15 separate slides per region in 3 mice per group ^&&^*p* < 0.01 and ^&&&&^*p* < 0.0001 vs. young Tg-SwDI thalamus and cortex, respectively. **(C)** Representative T2*MRI images of cortex and thalamus of Tg-SwDI mice 6 and 18 months old. Arrows indicated the existence of small microbleeds in both regions. **(D)** Box and whisker plot of the total number of cerebral microbleeds (CMBs) detected on MRI in Tg-SwDI mice (6 and 18 months old) and age-matched control (WT) mice. Many CMBs were detected in Tg-SwDI mice (6 and 18 months old) with significantly more in the old Tg-SwDI group. More occasional CMBs were found in old control WT mice, but not in young WT mice. *N* = 10 mice per group **p < 0.01 and ***p < 0.001 comparing age-matched mice (6 and 18 months old); ^&&&^*p* < 0.001 comparing to Tg-SwDI (6 months old). Inset: Representative image for histological verification (Prussian blue staining) of a CBM (scale bar 50 μm). **(E)** Stacked bars graph represents brain distribution of the CBMs in all experimental groups. (*n* = 5 mice per group). **(F)** MRI analysis of the BBB permeability in Tg-SwDI mice (6 and 18 months old) and corresponding age matched controls. Representative of images of Gd-DTPA enhancement in T1w MRI and T1 map of blood-brain barrier transfer coefficient *K*_*i*_ varying from 0 to 1 min^–1^. **(G)** Violin graphs of blood-brain barrier transfer coefficients (*K*_*i*_; min^–1^) in thalamus and cortex, the two most affected regions, in all experimental groups. *n* = 10 mice per group; *p < 0.05 comparing with control; ^&&^*p* < 0.01 comparing young Tg-SwDi (6 months) with old Tg-SwDi (18 months). **(H)** Influx rate constant (*K*_*i*_) for sodium fluorescein (SF, 376 Da), Inulin (5 kDa), and dextran (20 kDa) in the whole brain in young (6 months) and old (18 months) Tg-SwDI and control (wildtype) mice. Values are means ± SD, *n* = 10. *p < 0.05 and **p < 0.01 vs. corresponding aging control; ^&&^*p* < 0.01 vs. young Tg-SwDI.

Cerebral amyloid angiopathy vascular pathology is characterized by progressive vascular Aβ deposition, microbleeds, and persistent BBB hyperpermeability. In a murine CAA model, transgenic mice with Swedish Dutch Iowa mutation (Tg-SwDI) in Aβ precursor protein, the kinetics of the appearance of these three signs showed some differences. Vascular Aβ deposition begins to appear at ∼6 months in these mice with progressive accumulation and coverage of capillaries in the cortex (1,404 ± 422 μm^3^) and in the thalamus (1271 ± 428 μm^3^) by 18 months (Figures 1A,B). Thus, young adult Tg-SwDI mice (6 months) showed only sporadic Ab accumulation in both cortex and thalamus, which is significantly lower than aging Tg-SwDI mice (*p* < 0.0001 cortex and *p* < 0.01 thalamus; Figure 1B).

The second pathological sign of CAA, cerebral microbleeds (CMB), was evaluated by MRI T2* scan. CMBs were predominantly distributed in the cortex and thalamus (7 ± 3 and 8 ± 2 CMBs, ∼56% of total number), with a further ∼19% in the cerebellum and sporadic occurrence in the hippocampus in (18 months old) Tg-SwDI mice (Figures 1C–E). CBM size on MRI was mostly between 0 and 100 μm^3^, with the majority being less than 50 μm^3^ (9 ± 2 CMBs, 6 months, and 20 ± 4 CBMs, 18 months), while 50–100 mm^3^ CMBs were present predominantly in aged Tg-SwDI mice (10 ± 4, compared to 2 ± 1 at 6 months). Occasional CBMs larger than 100 μm^3^ were detected in aged Tg-SwDI mice (data not shown). Taking into consideration age, 6 months vs. 18 months old mice, the total number of microbleeds significantly increased with age and Aβ deposition around vessels (9 ± 2 in 6 months and 27 ± 6 CMBs in 18 months old Tg-SwDI mice; Figures 1C,D). Occasional CMBs were found in aged (18 months) but not young (6 months) WT controls. Overall, these results indicate that aging and Aβ accumulation have an enhancing role in the occurrence of CBMs.

Blood–brain barrier permeability was examined in the context of aging and brain region by MRI. A contrast agent, Gd-DTPA, was administered, and T1MRI pre- and post-scanning were used to evaluate the magnitude and regional specificity of BBB leakage. As shown in Figures 1F,G, the predominant regions of intense BBB leakage were in the cortex and thalamus. The influx rate constant, *K*_*i*_, for the movement of contrast agent Gd-DTPA from blood to brain was increased in young (6 months) and old (18 months) Tg-SwDI mice compared with aged, matched WT mice in both thalamus and cortex (*p* < 0.05). There was also significantly greater (*p* < 0.05) BBB disruption in old vs. young Tg-SwDI mice in the thalamus (Figure 1G). It should be noted that there was also some BBB hyperpermeability in aged WT mice compared to young WT mice (Figure 1G). Different sized tracers (MW range: 376 Da–20 kDa) were used to examine the size selectivity of BBB permeability using an *in vivo* permeability assay. Compared to age-matched WT mice, young TgSwDI mice showed increased (*p* < 0.05) permeability for small size tracers (sodium fluorescein, SF, MW = 376 Da, and inulin MW = 5 kDa) but not a larger molecular weight tracer (dextran 20 kDa). However, old Tg-SwDI mice showed enhanced BBB permeability for all tracers ranging from SF (376 Da) and inulin (5 kDa) (*p* < 0.01) to 20 kDa dextran (*p* < 0.05) when compared with age-matched control and young Tg-SwDI mice (*p* < 0.01) (Figure 1H). As in the MRI assessment, there was increased BBB permeability to SF (376 Da, *p* < 0.01) and inulin (5 kDa, *p* < 0.01) tracers in aged compared to young WT mice.

**FIGURE 2:**
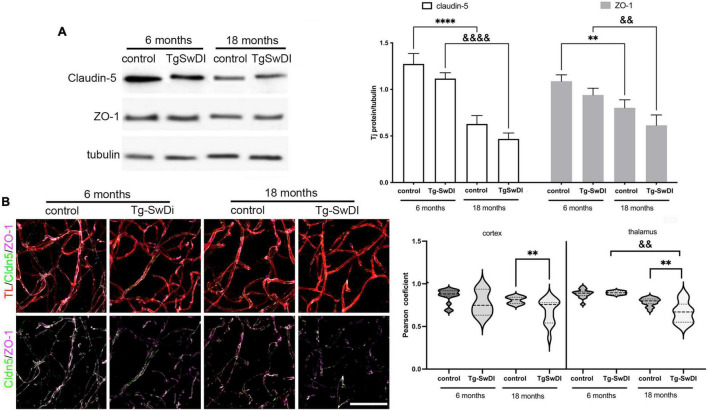
**(A)** Western blot and semi-quantitative densitometric analysis of claudin-5 and ZO-1 protein expression in microvessels isolated from young Tg-SwDI (6 months) and old Tg-SwDI age (18 months) mice and corresponding age-matched control. Protein levels were normalized to tubulin. Notice the decreased expression of claudin-5 and ZO-1 in total protein expression in Tg-SwDi (18 months old) as well in aging mice (18 months old). Graph represents means ± SD, *n* = 5; **p < 0.01, ^&&^*p* < 0.01 and ***p < 0.001, ^&&&^*p* < 0.001 comparing genotypes of different ages. Western blotting image is one of five independent experiments. **(B)** Representative images of the maximum 3D projection of z-stack (400, 400, and 200 μm) after immunofluorescence for tight junction proteins claudin-5 (green) and ZO-1 (Far red) in the cortex of young Tg-SwDi (6 months) and old Tg-SwDI age (18 months) mice and corresponding age-matched wildtype controls. Blood vessels were labeled with Tomato lectin (TL; red). Scale bar 50 μm. Arrow indicates the colocalization site of claudin-5 and ZO-1. Semiquantitative analysis of the colocalization of claudin-5 and ZO-1 in microvessels as a measure of claudin-5/ZO-1 interaction by calculating a Pearson correlation coefficient in cortex and thalamus in all experimental groups. The Tg-SwDI (18 months) showed a decreased level of the claudin-5 and ZO-1 colocalization compared with age-matched control in cortex and thalamus, as well as with young Tg-SwDI (6 months) in the thalamus. *N* = 3 mice per group and a total of 15 slides for both regions, **p < 0.01 compared to control and ^&&^*p* < 0.01 compared to the same genotype. ****Comparing to control and ^&&&&^comparing to same genetype.

This increased BBB permeability with CAA and aging were associated with changes at the tight junction (TJ) complex and, particularly, in alterations in the major occlusion protein, claudin-5, as well as the TJ scaffolding protein ZO-1 (Figure 2). At the protein level (Western blot), aging markedly reduced claudin-5 and ZO-1 expression in both Tg-SwDI and WT mice (Figure 2A), which may be associated with the increased BBB permeability with age in both types of animals. However, there were no significant differences between Tg-SwDI mice and corresponding age-matched controls (Figure 2A). Due to the importance of claudin-5 and ZO-1 interaction for the stability of TJ complex and brain endothelial barrier integrity, we assessed claudin-5/ZO-1 colocalization in blood vessels in Tg-SwDI and WT mice. The colocalization of claudin-5 and ZO-1 was significantly decreased in cortical and thalamic blood vessels in Tg-SwDi mice (18 months old) compared to age-matched WT mice (*p* < 0.01). Intriguingly, the most profound decreased protein expression and colocalization of claudin-5 and ZO-1 was seen in thalamic blood vessels, a region of intense BBB leakage (Figure 2B). Overall, the compromised BBB integrity in developing CAA may involve the interaction between an age-dependent reduction in TJ proteins and an Aβ-dependent disruption in TJ organization.

### Transcriptional Profiling of Blood–Brain Barrier Injury in Cerebral Amyloid Angiopathy

Amyloid-β deposition is considered a critical factor in disrupting BBB integrity and fueling processes associated with brain injury in CAA. The profound effect of Aβ deposition is seen at the functional level as BBB hyperpermeability and increased the occurrence of microbleeds. However, in the absence of microvascular Aβ deposition, Tg-SwDI mice still had increased BBB permeability, and microbleeds sporadically occurred. This indicates that events associated with brain endothelial cell dysfunction could be critical in CAA pathogenesis. To profile brain endothelial cell dysfunction in CAA, RNA seq analysis of isolated microvessels was performed to address the following questions: (a) whether the amyloid deposition has profound or causative effect on brain endothelial cell dysfunction, (b) what is the profile of the brain endothelial cells that lead to early BBB permeability and microbleeds focusing on endothelial cell dysfunction as a primary source of pathological changes in CAA.

**FIGURE 3:**
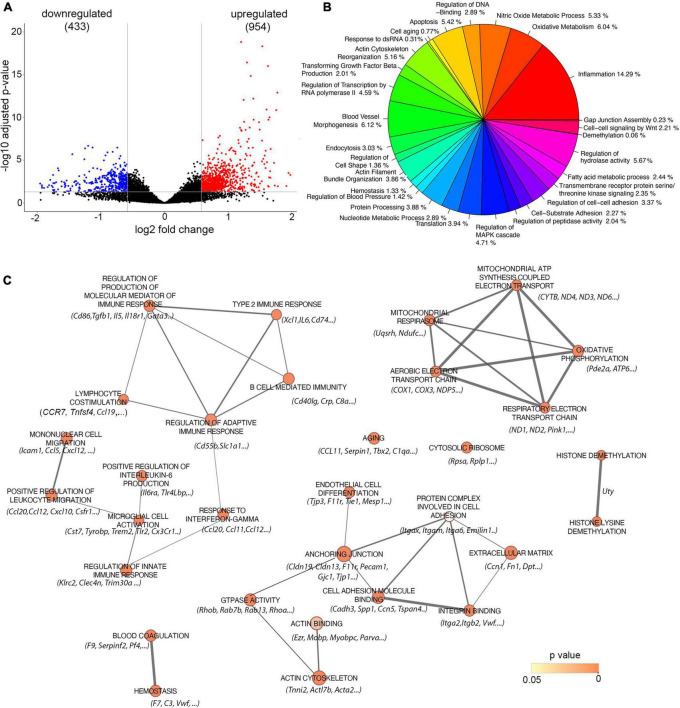
Analysis of Gene expression in isolated microvessels of Tg-SwDI (18 months) and control (18 months). **(A)** Volcano plot represents differential expressed genes (DEGs). The *x* and *y* axes show log2 fold change and −log10 *p*-value, respectively. Red points are up-regulated genes, blue points are down-regulated genes, and black points are genes with no significant difference. **(B)** Distinct transcriptomic profiles of 18 months old Tg-SwDI mice with fully developed CAA pathology. Pie chart represents a summarized gene ontology (GO) enrichment analysis of all three categories: biological processes, molecular function, and cellular compartment of differentially regulated genes. Only GO terms enriched with a *p* < 0.05 were selected and summarized (see methods). **(C)** Enrichment Map visualization of the top 30 ranked GSEA results obtained from analysis between Tg-SwDI (18 months) and control (18 months). Nodes represent enriched GO terms, and edges represent overlapping relations between GO terms. Nodes are colored and color intensity is proportional to *p*-value significance. Since conservative thresholds were used to select gene-sets, most of the node colors are intense (corresponding to highly significant gene-sets). The thickness of edges represents the richness of shared genes between connected nodes between each gene sets. Top listed genes are included.

To analyze the amyloid effect on brain endothelial cell dysfunction, barrier hyperpermeability, and microbleeds, we analyzed the transcriptional profile of microvessels isolated from Tg-SwDI mice with Aβ deposition (aged 18 months) and compared it to age-matched control WT mice without amyloid deposition. We identified 1,387 differentially expressed genes (DEGs) defined by fold changes ± 1.5 and false discovery rate adjusted on *p* < 0.05. Of those genes, 954 were upregulated while 433 downregulated in Tg-SwDI (18 months old) compared to the age-matched control group (Figure 3A).

The general overview of the GO ontology data revealed that 14% of enriched genes are associated with inflammation (e.g., inflammatory response, macrophages, leukocyte activation and chemotaxis, and regulation of inflammatory process), 6% with oxidative metabolism and mitochondrial damage (oxidative phosphorylation, cellular respiration), 5% with nitric oxide metabolic process, 6% with endothelial cell (blood vessel) morphogenesis (cell junction, cell-substrate interaction, extracellular matrix, angiogenesis), 5% with apoptosis, and 5% with actin cytoskeletal organization (Figure 3B).

The GO enrichment analysis was further performed to analyze the profile of genes related to amyloid-associated vascular pathology in CAA. The network map parameters were set up on the cut-off values: *p* < 0.05, FDR *q* < 0.05, and overlap index > 0.3. As shown in Figure 3C, significant enrichment of the DEG is present in 30 GO terms that mainly belong to inflammatory response, oxidative metabolism, and mitochondrial alterations, as well as endothelial/blood vessel morphology. The significantly enriched GO groups are lymphocyte co-stimulation (NES = 2.040, *p* = 0.00025) with prominent altered genes TNFSf4, CD274, and CCL19; type 2 inflammatory response (NES = 2.092, *p* = 0.00025) with significantly upregulated genes XCL1, CD74, and IL6; B-cell-mediated immunity (NES = 1.923, *p* = 0.00029) with a marked change in the CD40Lg gene. Upregulation and high enrichment of DEG were also present in a cluster of genes that regulate response to interferon-γ (NES = 2.303, *p* = 0.000291) with prominent altered genes including CCL11, CCL20, CCL12, and Tlr2. The innate immune response (NES = 2.073, *p* = 0.0003) and macrophage/microglia cell activation (NES = 2.073, *p* = 0.00025) are equally represented in response to amyloid deposition around vessels. Three genes are directly associated with CAA and AD pathology and they significantly upregulated cystatin7 (Cys7), TREM2, Tyrobp and Tlr2. These genes/proteins are involved in the initial general immune response and microglial activation (Figure 3C).

A high DEG enrichment score and significant upregulation also occurred in the oxidative phosphorylation cluster, including the following pathways: aerobic electron transport genes (NES = 2.228, *p* = 0.00025), mitochondrial ATP synthesis coupled electron transport (NES = 2.174, *p* = 0.00026), and respiratory electron transport chain (NES = 2.185, *p* = 0.00027), which involved Cox-1, -2, and -3; Cytb; and NADH- oxidoreductase ND4, ND3, ND6, and ND1 as prominent altered genes (Figure 3C).

High enrichment of genes and significant upregulation were also present in the group involved in endothelial development (NES = 2.225, *p* = 0.00026) with associated genes Itgax, F11r (JAM-A), and CXCL10. Another significantly enriched and upregulated cluster of DEG was blood coagulation, with associated genes for Serping1 and Serpin 1, platelet factor-4 (PF4), the complement cascade (C3, C1aq), aging (NES = 1.812; *p* = 0.00030), and representative gene CCL11, as well genes involved in impaired ribosomal biogenesis (NES = 1.778; *p* = 0.0031). In addition, notable changes were also present in two GO groups: actin cytoskeleton (NES = 1.719; *p* = 0.00043) and cell–cell junction (NES = 1.470, *p* = 0.0013), with prominent changes in the following genes Acxtb4, Tnnt3, Ezrin, Tjp3, Tjp1, F11r, Gjc1, genes that regulate junction assembly, Rap1b, Rab13, Trpv, and cell polarization Amotl1, Pard3b, and Podxl (Figure 3C).

Overall, the transcriptomic profile of the CAA microvasculature is shifted to a proinflammatory phenotype with elements of both adaptive and innate immune response, with particularly high macrophage/microglial activation. Amyloid deposition causes intense endothelial cell and barrier dysfunction with impairment of the mitochondrial respiratory chain, profound senescence of cells, changes to pro-coagulation phenotype, remodeling of barrier properties due to cell junction and actin cytoskeleton reorganization, and changes in cell polarity (Figure 3C).

**FIGURE 4:**
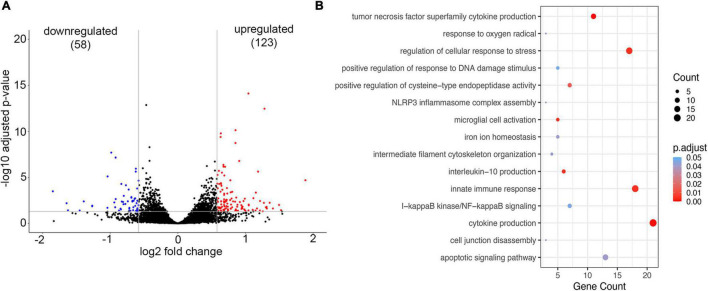
Analysis of Gene expression in isolated microvessels of Tg-SwDI (6 months) and control (6 months). **(A)** Volcano plot represents differential expressed genes (DEGs). The *x*- and *y*-axes show log2 fold change and −log10 *p*-value, respectively. Red points are upregulated genes, blue points are downregulated genes, and black points are genes with no significant difference. **(B)** The scatter plot of GO-based overrepresentation analysis results of DEGs in Tg-SwDI (6 months). The *y*-axis shows significantly (*p* < 0.05) overrepresented GO terms. The *x*-axis shows the gene count number of genes enriched in each GO term.

To establish whether brain endothelial cells have a “preexisting” condition for developing CAA vasculopathy, the transcriptional profile of the microvessels from young Tg-SwDI mice (6 months old) was compared to age-matched WT mice (6 months old). We detected a total of 181 DEGs in blood vessels isolated from young Tg-SwDI mice of which 123 DEGs were significantly upregulated and 58 downregulated (*p*-adjusted < 0.05; Figure 4A). The GO-based over-representative analysis (cut-off *p*-adjusted < 0.05) revealed that DEG was associated with inflammatory cytokine production (*p*-adjusted = 0.00025), tumor necrosis factor superfamily of cytokines production (*p*-adjusted = 0.00029), microglial activation (*p*-adjusted = 0.00469), innate immune response (*p*-adjusted = 0.00046), NLRP3 inflammasome complex assembly (*p*-adjusted = 0.039), and I-kappa B kinase/NF-kappa B signaling (*p*-adjusted = 0.0467). Genes common in these categories were TREM2 and its acting partner Tyrobp, TREM32, TLR2, complement C1qb, and CD84. All of these genes are involved in microglial activation and innate immune response, regulating cytokine production (e.g., IL6 and IL10). In the GO pathway “cell response to stress” (*p*-adjusted = 0.00464), Ezr (ezrin) and Parp9 (mono-ADP-ribosyltransferase) were highly upregulated, both of which are involved in actin cytoskeleton alterations and BBB disruption (Figure 4B). There was also over-representation in the GO pathway “response on oxygen radicals” (*p*-adjusted = 0.0411), with marked upregulation of the gene Nfe2l2 that encodes transcription factor NRF2, a regulator of antioxidant protein expression that protects against oxidative damage triggered by inflammation or injury (Figure 4B). This suggests that inflammation and alterations in oxidative metabolism could be an underlying cause of BBB dysfunction and microbleeds in the early stage of CAA vasculopathy when there is very low Aβ deposition.

**FIGURE 5:**
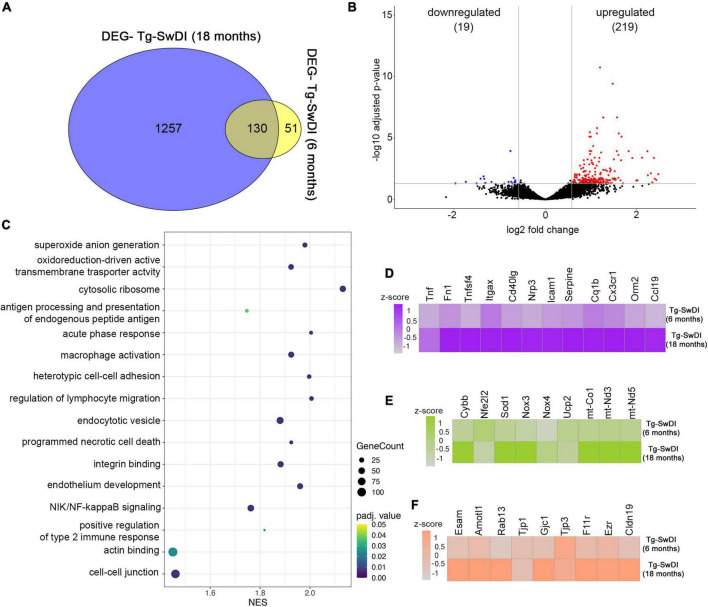
Analysis of Gene expression in isolated microvessels of old Tg-SwDI (18 months) and young Tg-SwDI (6 months). **(A)** Venn diagram showing the number of DEGs among Tg-SwDI 6 months and 12 months old. Values in the intersecting regions represent the number of overlapped DEGs between the comparisons, and values in the non-intersecting regions represent the number of DEGs that were unique to the corresponding comparison. **(B)** Volcano plot shows differentially expressed genes (DEGs) in Tg-SwDI (18 months) compared with Tg-SwDI (6 months). Red points are up-regulated genes, blue points are down-regulated genes, and black points are genes with no significant difference. The *x*- and *y*-axes show log2 fold change and −log10 *p*-value, respectively. **(C)** Gene set enrichment analysis demonstrates the top 20 enriched GO terms in Tg-SwDI (18 months) compared with Tg-SwDI young (6 months). The listed gene sets belong to all GO categories (biological process, cellular compartment, and molecular function). NES, normalized enrichment score. The node size represents the gene count. The color intensity represents *p*-adjusted value < 0.05. **(D–F)** Heat maps show the genes differentially expressed in microvessels isolated from Tg-SwDI (18 months) compared with Tg-SwDI (6 months). Heat maps for the differential expression of genes related to inflammation **(D)**, oxidative metabolism **(E)**, and junction proteins **(F)** are shown. The color scale represents the row *z*-scores of normalized gene counts.

We further analyzed potential markers that lead to exacerbation of CAA vasculopathy by comparing transcription profiles in old vs. young Tg-SwDI (18 vs. 6 months). Young Tg-SwDI had a total of 181 DEGs, while old Tg-SwDI mice had 1,381 DEGs with 131 overlapping genes. A total of 51 DEGs were present only in the young and 1,257 only in the old Tg-SwDI mice (Figure 5A). Shared DEGs included microglial activation factors Tlr2, Trem2, Tyrobp, Itgax, C1qb, cytoskeleton protein Ezrin (Ezr), and the mitochondrial Uncoupling protein 2 (UCP2) involved in reducing oxidative stress. Comparing these two groups, we have identified 238 DEGs of which 219 are upregulated while 19 are downregulated in the old Tg-SwDI group (Figure 5B). GO enrichment analysis was used to identify microvascular enriched genes in three categories: biological processes, cellular compartment, and molecular function with a set up on the cut-off values, *p* < 0.05 and FDR *q* < 0.05 to analyze DEGs in old and young Tg-SwDI mice. This identified the top ten biological processes significantly enriched in 18 months old Tg-SwDI mice: acute phase response (NES = 2.004, *p* = 0.00021), regulation of type 2 immune response (NES = 1.921, *p* = 0.000021), antigen processing and presentation of exogenous antigen NES = 2.1693, *p* = 0.00022), superoxide anion generation (NES = 1.980, *p* = 0.00022), heterotypic cell–cell adhesion (NES = 1.997, *p* = 0.00022), regulation of lymphocyte migration (NES = 2.006, *p* = 0.00021), NIK/NF-kB signaling (NES = 1.937, *p* = 0.00024), and macrophage activation (NES = 1.925, *p* = 0.00023). Regarding cellular components, the most enriched area were as follows: cytosolic ribosome (NES = 2.132, *p* = 0.00023) with upregulated ribosomal protein Rps3 transcript associated with NFκB activation and rapid cellular activation response; oxidoreductase complex (NES = 1.915, *p* = 0.00023); endocytic vesicle (NES = 1.880, *p* = 0.00025) that includes Ap2s1, WAS, Rab7b LAMP, Rab13, which control clathrin-dependent internalization, actin cytoskeleton regulation, trafficking from trans-Golgi to cell surface, and late endosomes and lysosome activity; cell–cell junction (NES = 1.461, *p* = 0.00029) with upregulation of structural proteins like Cldn19, F11r, Tjp1, Gjc1, Cdh5, polarity protein Amotl1, and regulatory protein Rap1. Among the molecular processes, significantly increased in Tg-SwDI 18 months old mice were: the integrin binding process (NES = 1.882, *p* = 0.00024) with significant upregulation of Itgb2, Itga6, Itgb4, Itgb4, and their binding partners; actin-binding pathway (NES = 1.451, *p* = 0.0017) with upregulation Parvin Alpha (PARVA), Ezrin (Ezr), and chemokine activity (NES = 2.215, *p* = 0.00022) in old Tg-SwDI (Figure 5C).

**FIGURE 6:**
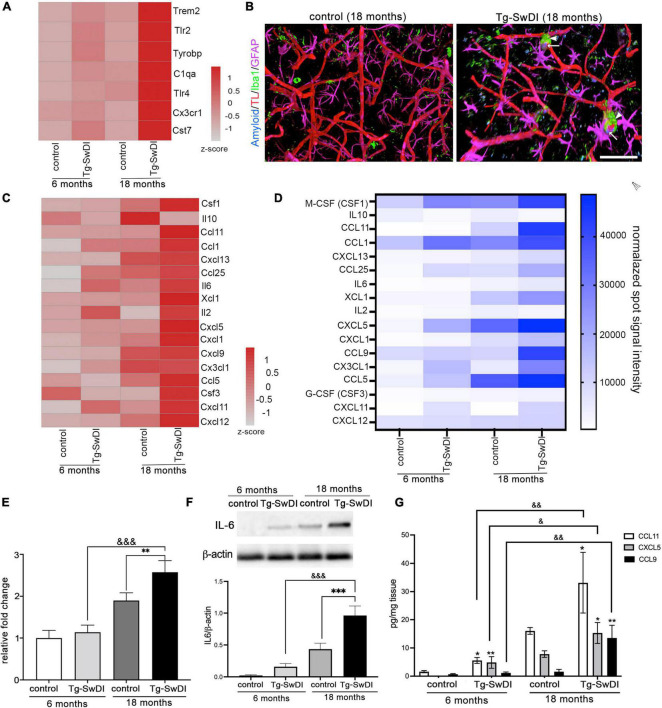
The inflammatory profile of brain microvessels in CAA vasculopathy. **(A)** Heat map of differentially expressed genes related to microglial activation in microvessels in all experimental groups. The color scale represents the row *z*-scores of normalized gene counts. **(B)** Representative images of the maximum 3D projection of z-stack (400, 400, and 200 μm) with immunofluorescence for astrocytes (GFAP, Far Red) and microglia (Iba1, green) in the cortex of Tg-SwDI (18 months) and corresponding control mice. Blood vessels were labeled with Tomato lectin (red) and Aβ with Amyloid Glo (blue). Scale bar = 100 μm. **(C)** Heat map of the part of differentially expressed genes related to chemokine and cytokines in all experimental groups. The color scale represents the row *z*-scores of normalized gene counts. **(D)** Heat map of the inflammatory protein array from all experimental groups. The selected cytokines/chemokines verify the altered gene expression identified in RNA seq analysis. **(E–G)** Verification of alterations in mRNA and protein expression for select genes. **(E)** Real-time RT-PCR confirmed ICAM-A upregulation in Tg-SwDI mice as well as in old WT mice. Values are means ± SD, *n* = 3. **p < 0.01 vs. corresponding aging control; ^&&&^*p* < 0.001 vs. young Tg-SwDI. **(F)** Western blot and semi-quantitative densitometric analysis of IL-6 protein expression in microvessels isolated from young Tg-SwDI (6 months) and old Tg-SwDI (18 months) mice and corresponding age-matched controls. Protein levels were normalized to β-actin. Graph represents means ± SD, *n* = 3; ***p < 0.001 comparing Tg-SwDI to age matched control and ^&&&^*p* < 0.001 comparing genotypes of different ages. Western blotting image is one of three independent experiments. **(G)** Chemokines CCL11, CCL9 and CXCL5 protein level in brain microvessels evaluated by ELISA in all experimental groups. Values are means ± SD, *n* = 3. *p < 0.05 and **p < 0.01 vs. corresponding aging control; ^&^*p* < 0.05 and ^&&^*p* < 0.01 vs. young Tg-SwDI.

A major difference between old and young Tg-SwDI mice is a profound inflammatory response with some of the key inflammatory mediators highly upregulated in old compared to young Tg-SwDI mice. This includes regulators of the adaptive immune response (e.g., CD40lg, XCL1, Tnfsf4, and ICAM-1) associated with T and B cell activation, mobilization, and chemotaxis (Figure 5D). In addition, there was high expression related to the innate immune response including the acute response protein Orosomucoid 2 (Orm2), complement activation cascade C1qb, fibronectin-1 (Fn1), and chemokine CCL19 and adhesion molecules (ICAM-1; Figures 5D, 6E). A second category represented genes involved in superoxide anion generation (Cybb, Nox3, Cox1, Sod1, Cox3, mt-ND5, mt-ND3) with the downregulation of two protective regulators of oxidative metabolism, Ucp2 and Nfe2I2 (Figure 5E). The third category with altered transcriptomics were genes involved in cell junction interactions, including tight junction proteins Tjp1, Cldn19, and F11r; organization of cell polarity and junction Amotl1, Rab13, and Podxl; gap junctions Gjc1 and Gja1; adherens junction Cdh1 and Cdh5; cytoskeletal organization ezrin (Ezr); interaction of cells with extracellular substrate of platelets Itgax, Pecam, and Pf4 (Figure 5F). These data indicate profound alterations in the microvascular transcriptomic profile induced by amyloid deposition and aging, underlying most of the typical signs of CAA vasculopathy.

One key regulator of processes at the microvasculature in CAA is inflammation. In particular, changes were seen in a group of genes associated with microglial activation including Cst7, Trem2, TLR2, Tryobp, Cx3Cr1, and C1qa in aged Tg-SwDI mice (Figure 6A). Intriguingly, young Tg-SwDI mice also had alterations/upregulation in this set of genes compared to WT mice (young and old), indicating that microglial priming in CAA could be critical in BBB pathogenesis. This transcriptional profile was associated with morphological changes, with an increased presence of microglia in close proximity to those blood vessels with Aβ deposition which formed “cuff-like” structures in elderly Tg-SwDI mice (18 months; Figure 6B). This could indirectly be associated with the upregulation of microglia activation factors. There was transcriptome upregulation of T-cell-associated chemokines XCL1, CXCL12, CCL1, and CCL11; B-cell-associated activator and chemokines IL6, CXCL13, CCL19; and macrophages and microglial attractants CCL9, CCL5, CX3CL1, and cytokine CSF1 (Figure 6C). A protein array, Western blot, and ELISA were further used to confirm that cytokines/chemokines mRNA differences were reflected in altered protein levels (Figures 6D,F,G). Noticeable was an increased protein expression of IL-6, CCL9, CCL11, and CXCL5 in old TgSwDI mice as well as in aged WT mice (Figures 6F,G).

## Discussion

The vascular component of neurodegenerative diseases has gained more attention in recent years due to evidence that it can directly affect and facilitate cognitive decline and pose a risk for the occurrence of stroke. CAA vasculopathy is commonly associated with AD, sharing a common pathological substrate, Aβ deposition. Although emerging evidence has revealed some overlap and interconnection between Aβ accumulation and injury in the brain parenchyma and vasculature, the mechanisms of the CAA vasculopathy are still largely unknown. The present study identified the specific microvascular transcriptional profile in CAA vasculopathy associated with the appearance and degree of CAA-specific pathological signs (Aβ deposition, CMB, and BBB hyperpermeability) revealing potential causes of CAA pathology. The results highlighted that: (a) Aβ has a critical role in triggering brain endothelial cell and BBB dysfunction in CAA vasculopathy, causing an intense proinflammatory response, impairing oxidative metabolism, altering the coagulation status of brain endothelium, and remodeling barrier properties; (b) Aβ induced both adaptive and innate immune responses with macrophage/microglial activation and specific chemokines profile that support T and B cell transmigration; (c) early inflammation, particularly microglia/macrophage activation and mediators of B lymphocyte activity, may underlie CMBs and BBB hyperpermeability in the early stage of CAA vasculopathy. These findings are discussed below.

The development of CAA vasculopathy is associated with neurovascular dysfunction, BBB leakage, and persistent vascular and parenchymal inflammation, eventually leading to neurodegeneration (Viswanathan and Greenberg, 2011; Tanifum et al., 2014). The currently accepted pathogenesis indicates that inadequate clearance of Aβ favors the accumulation of neurotoxic Aβ oligomers in the brain interstitial fluid and at the BBB (Rosas-Hernandez et al., 2020; van Veluw et al., 2020). Aβ oligomers could have mechanical effects reducing capillary blood flow (hypoperfusion and hypoxia) and direct toxic effects damaging endothelial cells (Carrano et al., 2011; Okamoto et al., 2012; van Veluw et al., 2020). The response of the endothelial cells is denoted as proinflammatory, prompting a local perivascular inflammatory response that can trigger a vicious cycle of diminished brain endothelial cell Aβ-clearance, accelerating further endothelial injury and intensifying perivascular inflammation. Numerous studies have pinpointed amyloid-induced inflammation as a driving force in vascular and BBB damage in AD and CAA vasculopathy (Zhu et al., 2020; Antolini et al., 2021; Sakai et al., 2021). In general, amyloid-induced inflammation mostly involves an innate immune response featuring microglial activation, increased production of proinflammatory cytokines (TNF-α, IL-6, IL-1β, IL-1, IFN-γ, and IL-12) and chemokines (MIP-1α, MIP-1β, MCP-1, CCL-20, and CXCL-2), and infiltration of immune cells (monocytes and neutrophils) noticeable in the brain and CSF of patients with AD with CAA or CAA only, as well as in corresponding murine models (Vukic et al., 2009; Park et al., 2013; Wang et al., 2014; Scholtzova et al., 2017; Bowman et al., 2018; Robison et al., 2019; Sakai et al., 2021; Panitch et al., 2022).

Brain endothelial cells are endowed with highly restrictive barrier properties, reflected in specific junctional complex organization and high polarization, that help protect the brain. However, brain endothelial cells are dynamic, responding to extracellular environmental changes and playing a meaningful role in immune system function, acting as immune/inflammation effectors and immune cell mobilizers. Participating in the immune response, brain endothelial cells can induce cytokine production by immune cells (Mai et al., 2013; Cohen et al., 2017; Propson et al., 2021). Endothelial cells function as immune regulators either by activating or suppressing immune cell function, acting as conditional innate immune cells (Mai et al., 2013). In CAA vasculopathy, amyloid deposition triggers a robust inflammatory response, particularly related to innate immunity. Our transcriptional analysis revealed that Aβ induced the expression of innate immune receptors, including Toll-like receptors (Tlr2 and Tlr4), which activate intracellular inflammatory pathways mediated through NF-κB and the MAP kinases, IL1R21, a member of Toll-like receptor superfamily, IL-6, a variety of chemokines (CXCL1, CXCL5, CCL11, CCL12, CCL9, CCL5) as well as C1qb and C1qa, augmenting the intrinsic proinflammatory response and tailoring the brain endothelial cell proinflammatory phenotype. This process at BBB was further enhanced by the recruitment of blood monocytes with increased expression of ICAM, VCAM, ESAM, and JAM-A (F11r) on endothelial cells and chemokines (CCL3, CCL4, CCL20, and CXCL2) that guide transmigration as well as migration and engagement of microglial cells around affected brain capillaries. As part of the innate immunity response at the brain microvasculature, microglia play a critical role. Marked Aβ deposition causes the activation of perivascular macrophages/microglia that can build “cuff-like” structures around the Aβ-affected vessels. Recent studies pinpoint the existence of endothelial/microglial crosstalk via CSF1/CSF-1R signaling that regulates BBB integrity and systemic macrophage recruitment to the brain and interaction via Icam1–Itagm that activates integrin signaling pathways in both cell types (Honarpisheh et al., 2020; Zhu et al., 2020; Delaney et al., 2021; Zheng et al., 2021). In addition, our results revealed the microvascular profile that favors the disease phenotype of microglial mirrored in the upregulation of TYROBP, TREM2, and Cst7 in both early and late stages of CAA vasculopathy. TYROBP is associated with ICAM–Itgav activation, modulating the inflammatory response by releasing pro-inflammatory cytokines in a chronic condition. TREM2 activity is mostly associated with late AD onset, and it is a signaling partner with TYROBP in potential microglial activation and migration. Cystatin-7 (Cst7) was recently identified as a cause of vascular injury in an inherited form of CAA vasculopathy (Island type mutation) exacerbating further microglial induced inflammatory remodeling of BBB (Ghiso et al., 1986).

In addition to the innate immune response, several recent studies have implicated mediators of the adaptive immune response in CAA pathogenesis. This is reflected in increased T and B cell infiltration into the AD brain that affects the inflammatory response and ongoing vascular impairment and could be the cause of the limited efficacy of immune therapy in Aβ clearance (Spani et al., 2015; Ferretti et al., 2016; Jorda et al., 2019; Gate et al., 2020). Aβ accumulation around the blood vessel generates a potentially potent milieu for T and B cell recruitment by increasing the presence of chemokines like CCL1, XCL1, CCL5, CCL19 involved in lymphotaxis. In addition, the microvascular showed increased expression of the T and B cell co-stimulated molecules CD40lg and TNFSF4. Both are members of the TNF superfamily and are well-known for their classical role in stimulating antigen-presenting cells. High expression of CD40L and CD40 has been reported in and around Aβ plaques in AD brain indicating their role in inflammatory response associated with Aβ plaque pathology (Calingasan et al., 2002; Laporte et al., 2006; Ait-ghezala et al., 2008). They act on the interface between the innate (microglial) and adaptive arms of the immune system (Tan et al., 1999; Ait-Ghezala et al., 2005). TNFSF4 expression is reported in endothelial cells regulating their interaction with T lymphocytes (Mestas et al., 2005). It is important to highlight that shift toward adaptive immunity is dependent on Aβ deposition around the microvasculature as the component of adaptive immune response is not present in the early-stage CAA vasculopathy (Tg-SwDI young mice). Thus, the innate immune response and particularly microglial activation may initiate vascular and BBB injury, while the adaptive immune response is dependent on Aβ deposition and contributes to further microvascular deterioration in CAA vasculopathy.

In addition to inflammation, substantial evidence indicates defects in mitochondrial function in various cells of the neurovascular unit, as well as in brain parenchyma in the early and late stages of AD and CAA (Carrano et al., 2011; Todd et al., 2016; Solesio et al., 2018). In the early stage of CAA, with little amyloid accumulation, there is upregulation of protective systems against oxidative radicals at the BBB. We have identified three important factors, nuclear factor erythroid 2-related factor 2 (Nrf2), transcription factor NFE2L2, and mitochondrial uncoupling protein 2 (UCP2), which regulate a cellular resistance to reactive oxygen species (Ding et al., 2019; Deng et al., 2020). However, in aged mice with profound CAA pathology, a specific profile of mitochondrial damage is present. This includes the upregulation of Cybb, Nox4, and Nox3 transcripts. The nicotinamide adenine dinucleotide phosphate (NADPH) oxidases (NOX) enzymes are multi-subunit protein complexes and membrane-bound proteins whose main function is to transfer electrons across the plasma membrane to molecular oxygen, which results in the generation of the superoxide anion and subsequently reactive oxygen species (ROS), including hydrogen peroxide (H_2_O_2_) and hydroxyl radicals (OH^–^) (Brandes et al., 2014). Cybb (Nox1), together with Nox2 and Nox3, interact with p22*^phox^* transmembrane protein along with the cytosolic organizer subunits (p47*^phox^*, NOXO1), activator subunits (p67*^phox^*/NOXA2, NOXA1, p40*^phox^xs*), and the G-protein Rac (Kawahara et al., 2005; Ueno et al., 2005; Brandes et al., 2014). NOX4 is constitutively active and requires only p22*^phox^* for activity (Zana et al., 2018). Upregulation of Nox1, Nox 4, and Nox5 occurs during brain endothelial aging, cerebral ischemia, and in the AD brain (Lee et al., 2020; Zhang and Li, 2020; Park et al., 2021; Shen et al., 2021). Other upregulated transcripts were SOD1 mt-CO1 mt-ND3 and mt-ND5 linked to oxidative damage in AD in several studies (Bitto et al., 2017; Kraja et al., 2019; Cieslik et al., 2020). These results pinpoint that Aβ has a profound effect on oxidative damage at the BBB. However, the early stage of CAA vasculopathy is characterized by an upregulation in cell defense mechanisms against oxidative radicals, which in the later stages of CAA is replaced by mediators of oxidative injury.

Finally, it is important to highlight some other phenotypic and structural changes in the microvasculature during CAA vasculopathy in mice. A hallmark of CAA vasculopathy is BBB hyperpermeability with altered expression of major TJ proteins, claudin-5, ZO-1, claudin-1, and occludin. Intriguingly, we did not find changes in claudin-5 or occludin at the mRNA level. In contrast, ZO-1 (Tjp1) showed alterations in transcriptome pattern in Tg-SwDI mice in both young and old compared to age-matched controls. It should be noted that TJ function is not only dependent on TJ mRNA and protein levels but also onlocalization and interactions between TJ proteins. Interestingly, we found that aged Tg-SwDI mice had disrupted claudin-5/ZO-1 interaction. Two other TJ proteins showed upregulation in aged Tg-SwDI mice, JAM-A (F11r), and ESAM. Both proteins have dual actions, acting as structural TJ proteins, but also as adhesion molecules controlling monocyte and T cell recruitment to the brain under inflammatory conditions (Wegmann et al., 2006; Stamatovic et al., 2012; Sladojevic et al., 2014). Considering the intense inflammatory response induced by Aβ, it is expected that the upregulation of JAM-A (F11r) and ESAM is associated with leukocyte transmigration in CAA vasculopathy.

In summary, brain endothelial cell dysfunction and a compromised BBB play critical roles in the pathogenesis of CAA that may lead to consequent brain injury and cognitive decline. Inflammation, particularly components of the innate immune response and microglial, is a major driving force triggering BBB injury and a vicious cycle involving amyloid accumulation, worsened inflammation, and brain endothelial cell metabolic dysfunction. Thus, treating the inflammatory response in the early stage of CAA and tailoring the anti-inflammatory strategy to modify both the adaptive and innate immune response in later stages could be an approach to prevent the progression of CAA vasculopathy at the BBB.

## Data Availability Statement

The datasets presented in this study can be found in online repositories. The name of the repository and accession number can be found below: National Center for Biotechnology Information (NCBI), https://www.ncbi.nlm.nih.gov/bioproject/, PRJNA833447.

## Ethics Statement

The animal study was reviewed and approved by the Institutional Animal Care and Use Committee, University of Michigan.

## Author Contributions

MS performed most of experiments, all bioinformatic analysis, and contributed to the writing of the manuscript. AC-M performed immunostaining and imaging analysis and contributed to the writing of the manuscript. SS contributed to MRI analysis, designing the study, and writing of the manuscript. RK contributed to designing the study and writing the manuscript. AVA designed the study, supervised the experiment, and wrote the manuscript. All authors contributed to the article and approved the submitted version.

## Conflict of Interest

The authors declare that the research was conducted in the absence of any commercial or financial relationships that could be construed as a potential conflict of interest.

## Publisher’s Note

All claims expressed in this article are solely those of the authors and do not necessarily represent those of their affiliated organizations, or those of the publisher, the editors and the reviewers. Any product that may be evaluated in this article, or claim that may be made by its manufacturer, is not guaranteed or endorsed by the publisher.
